# Predictors of disease alleviation with mandibular advancement devices in obstructive sleep apnea: a retrospective cohort study

**DOI:** 10.1186/s13005-025-00504-x

**Published:** 2025-04-14

**Authors:** Eva Paddenberg-Schubert, Benedikt Holmer, Sebastian Krohn, Helmut Hösl, Peter Proff, Christian Kirschneck, Michael Arzt

**Affiliations:** 1 Department of Orthodontics, University Hospital Regensburg, 93053 Regensburg, Germany; 2 Centre of Sleep Medicine, Department of Internal Medicine II, University Hospital Regensburg, 93053 Regensburg, Germany; 3 Department of Orthodontics, University of Bonn, 53111 Bonn, Germany

**Keywords:** Lateral cephalogram, Sleep disorder, Treatment success, Apnea-hypopnea index

## Abstract

**Background:**

Obstructive sleep apnea (OSA) can be treated with mandibular advancement devices (MAD), preventing collapse of the upper airway and decreasing apnea-hypopnea index (AHI)/ h. Disease alleviation is expected to vary depending on specific predictors including OSA-severity and cephalometric parameters. This retrospective cohort study aimed to identify predictors of disease alleviation with MAD in adult patients with mild to moderate and severe OSA. Secondary outcomes included assessing the necessity of lateral cephalograms and the therapeutic success in severe OSA-cases.

**Methods:**

OSA-patients, treated with MAD at the orthodontic department of the University Hospital Regensburg, Germany, were allocated to mild to moderate (AHI ≤ 30/ h) and severe OSA groups (AHI > 30/ h). BMI, poly(somno)graphic, demographic and cephalometric variables were evaluated before (T0) and after 3 to 6 months of MAD-treatment (T1). Applying linear regression analyses, predictors were identified, following an assessment of their effect on disease alleviation by independent two-tailed t-tests for continuous, and absolute and relative frequencies for categorical variables. Then, the need for cephalometric analysis and the disease alleviation in severe OSA-patients were evaluated.

**Results:**

Sixty-six predominantly male patients (mean age 55 ± 11 years; male:female = 52:14) were stratified to mild to moderate (*n* = 45) and severe (*n* = 21) OSA-groups. Regression analysis revealed baseline-AHI as a significant and relevant predictor, whereas few cephalometric parameters proved significance with small effect sizes (absolute AHI/ h-reduction, univariate model: -0.64 (95% CI: -0.75; -0.53), *p* < 0.001, R² = 0.666). Compared to mild to moderate OSA-cases, severe OSA-patients had a significantly higher AHI (19.1 ± 11.7 vs. 6.0 ± 4.0, *p* < 0.001) at T1, but also a higher disease alleviation according to absolute AHI-reduction (-26.1 ± 16.0 vs. -9.6 ± 6.4, *p* < 0.001), indicating comparable treatment success in all OSA-degrees.

**Conclusions:**

Disease alleviation with MAD in adult OSA-patients can be predicted with specific poly(somno)graphic parameters (especially baseline-AHI), whereas cephalometric variables appeared inappropriate. Still, lateral cephalograms are helpful in the diagnostics and follow-up of MAD-treatment, e.g. for evaluating side effects. Providing good patient selection, therapeutic success can be achieved in both severe and mild to moderate OSA.

**Supplementary Information:**

The online version contains supplementary material available at 10.1186/s13005-025-00504-x.

## Background

In obstructive sleep apnea syndrome (OSA) the upper airway is obstructed, resulting in an interruption of breathing. The diagnosis considers the apnea-hypopnea-index per hour of sleep (AHI/ h), measuring the events of complete obstruction (apnea) and decrease of the airflow by 50% (hypopnea) for at least 10 s. OSA is defined by AHI > 15/ h or AHI ≥ 5/ h combined with typical clinical symptoms or comorbidities, such as daytime sleepiness and cardiovascular diseases, respectively [1, 2]. It is associated with several risk factors like obesity, age and male sex [3]. The prevalence was reported to be 2 and 4% in femle and male adult patients, respectively [4], but a rise is observed in the general population [5, 6]. The increasing prevalence of OSA, severe clinical symptoms and accidents caused by patients concerned, clarify the need for disease alleviation with effective treatments.

Besides anamnestic variables and specific questionnaires [7], poly(somno)graphy is conducted to record parameters like the apnea index (AI)/ h, the oxygen saturation index (ODI)/ h, the AHI in supine position/ h, the ratio between central apnea (ZA) and all apneas (A) ZA/ A and the ratio between ZA and the combination of all apneas and hypopneas (AH) ZA/ AH. ODI/ h counts the events per hour, where arterial blood is desaturated by 3 or 4%,and is appropriate to screen for OSA [8]. OSA-diagnosis can be further classified, based on the AHI per hour: mild (AHI ≤ 15/ h), moderate (15/ h < AHI ≤ 30/ h) and high (AHI > 30/ h) OSA.

Treatment strategies for OSA-patients include, among others, therapy with continuous airway pressure (CPAP) or mandibular advancement devices (MAD). In MAD, an anterior movement of the mandible prevents a collapse of the upper airway. Compared to MAD, CPAP is regarded superior in terms of AHI-reduction in moderate and severe cases, whereas in mild forms both methods seem to be comparable [9–11]. Thus, MAD is an alternative treatment method in patients refusing CPAP [12], although in Germany such patients should have BMI < 30 kg /m² and AHI ≤ 30/ h [13]. Prior to MAD-treatment, further dental examinations are required to verify its indication. Here, lateral cephalograms can provide information about the upper airway space, the sagittal and vertical position of the jaw bases and the inclination of the incisors.

Although MAD is appropriate to reduce AHI even in the long-term [14], the effectiveness varies among individuals. Hence, it is of key interest to the practitioner to predict the expected success in advance. Several studies have looked for such predicting variables, but results in the literature are partly contradictory. For example, Becerra et al. found better treatment outcomes in patients with class II and mesiofacial or brachyfacial growth pattern than in cases with class I and a dolichofacial type [15]. However, this study was based on a small study collective (*n* = 22) and a different parameter than AHI-reduction to assess treatment effect. Another investigation reported no effect of dysgnathia on MAD-success, but identified age, neck circumference and baseline-AHI as factors associated with success rate [16]. Raunio et al., however, did not find a significant influence of age on treatment success [17].

Therefore, the primary aim of this retrospective cohort study was to identify predicting variables of disease alleviation with MAD in mild to moderate and severe adult OSA-patients by evaluating demographic, clinical, poly(somno)graphic and cephalometric variables. As secondary outcomes, we evaluated the need for lateral cephalograms for prediction and the therapeutic success as disease alleviation with MAD in severe OSA-cases.

## Methods

This study followed the declaration of Helsinki and is reported according to the STROBE guidelines. The participants were recruited retrospectively and analysed using pseud-anonymisation with a study number (approval number of the ethic’s committee 21-2259-104).

### Patients

All patients of the study cohort included were recruited from the department of orthodontics of the University Hospital Regensburg, Germany. All of them presented an OSA, which had been diagnosed by the Centre of Sleep Medicine of the department of Internal Medicine II of the University Hospital Regensburg, Germany, or by an external sleep-laboratory using polygraphic or polysomnographic examination. Polysomnography (PSG) is referred to as the reference standard in sleep diagnostics, providing comprehensive diagnostic data for detailed sleep staging, including neuronal activity, eye movements as well as cardiorespiratory parameters, sleep related noise (snoring/ teeth clenching) and muscle tone. PSG is used to diagnose a broad spectrum of sleep disorders, but due to its high level of effort there is a lack in availability. In contrast, polygraphy (PG) is a reduced but highly available method focusing on respiratory parameters such as airflow, oxygen saturation and respiratory effort. There is no EEG-based information for sleep stage analysis, but PG is typically employed for the evaluation of obstructive sleep apnea in patients with a high pre-test probability. There was no limitation applied in terms of the degree of severity of OSA.

Initially patients, who were treated between 06/2011 and 02/2021, were screened according to the inclusion and exclusion criteria. Only adult patients, aged at least 18 years, who presented an OSA as diagnosed by an AHI ≥ 5/ h and who received treatment with a MAD of the type “Thornton Adjustable Positioner” (“TAP^®^”) were included. Mandibular advancement devices (MAD) were used as a second-line treatment option, solely in cases with a medical indication, such as documented intolerance to CPAP therapy, which was in accordance with current clinical guidelines [12, 13]. Previous CPAP therapy was not mandatory, but body height and weight as well as baseline AHI and AHI during MAD treatment were required information for inclusion in the present study, whereas patients with incomplete data were excluded. Furthermore, patients receiving a combination of MAD and CPAP in parallel, were excluded from this investigation. Depending on the AHI/ h at baseline, participants were retrospectively stratified to two groups to distinguish between mild to moderate OSA (5 ≥ AHI/ h ≤ 30) and severe OSA (AHI/ h > 30).

### Assessments

All assessments occurred within clinical routine. Demographic data, sleep related questionnaires as well as BMI were taken from the patients’ records.

**Fig. 1:**
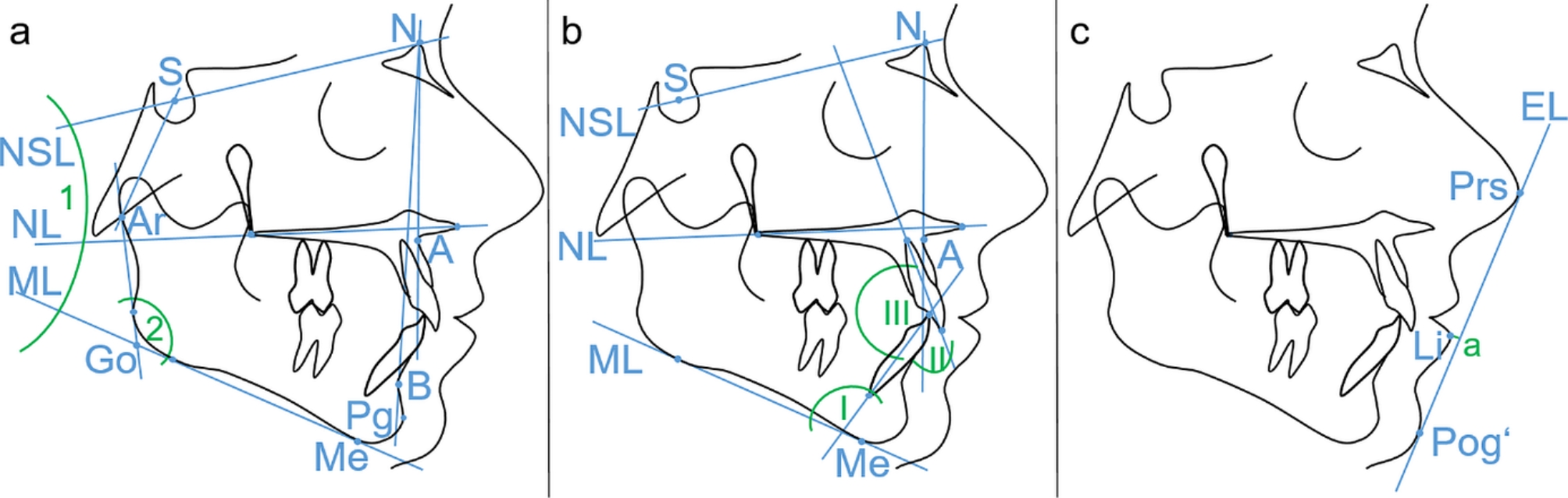
Relevant skeletal **(a)**, dental **(b)** and soft-tissue **(c)** cephalometric parameters evaluated. 1 = angle Nasion-Sella-Line (NSL)– Nasal Line (NL), 2 = Gonion angle, I = angle lower incisivus (UK1)– mandibular line (ML), II = angle upper inicivus (OK1)– line between Nasion and point A (NA), III = interincisal angle, a = distance Labrale inferius (Li)– Aesthetic Line (EL)

Only if the MAD-treatment was indicated from a dental point of view too, MAD-treatment was initiated. Briefly, patients had to fulfil the following criteria: protrusion of the mandible of at least 5 mm [18], no temporomandibular joint disorders, sufficient stability and amount of teeth providing support, no periodontitis and the absence of caries. During the process of recruitment all patients were clinically screened by using appropriate standardised screening protocols (https://www.dgfdt.de/richtlinien_formulare). The clinical protocol contained palpation of the masticatory muscles as well as the temporomandibular joint, examination of horizontal and vertical jaw mobility, investigation of static and dynamic occlusion and auscultation of potential joint sounds. Above all these objective diagnostics, assessment of functional anamnesis and absence of subjective symptoms was the basis for inclusion in the present study. Orthopantomograms were taken with the device Orthophos XG Plus (Sirona, Bensheim, Germany) at T0. Lateral cephalograms were routinely taken as part of the baseline and post-treatment (3 to 6 months, T1) diagnostics, although the last examination was not obligatory for all timepoints due to the retrospective study design. During the imaging patients presented an upright standing position with teeth in habitual intercuspation and the head adjusted and fixed in a cephalostat, ensuring the Frankfort horizontal plane to be parallel to the floor. All these radiographs were taken with the devices Orthophos XG 3D ready Ceph and Orthophos SL 2D (Dentsply Sirona, Bensheim, Germany), followed by an import of the digital images as lossless TIF-files into the software ivoris^®^ analyze pro (Computer konkret AG, Falkenstein, Germany) for cephalometric analysis, which was based on Segner and Hasund [19, 20]. Relevant cephalometric parameters are presented in Fig. 1.

Due to a potential correlation between posterior airway space and OSA [21, 22], lateral cephalograms were also used to determine the posterior airway space by performing six measurements PASP1 – PASP6, although the only relevant parameter was PASP1. It is defined as the distance between the posterior pharyngeal wall (pP1) and the point PNS (aP1), which is located at the height of the palatal plane (PP).

In the medical department, the following parameters were determined: age, sex, body height and weight, AHI/ h, AI/ h, ODI/ h and AHI in supine position/ h, ZA/ A and ZA/ AH.

All participants of this study received the MAD TAP^®^. After 3–6 months of MAD treatment (T1), patients underwent follow-up polygraphy or polysomnography at the Centre of Sleep Medicine or an external sleep-laboratory to reassess the clinical parameters recorded at T0.

At T1, in most cases, an additional lateral cephalogram without the MAD in place was performed for cephalometric analysis, following the same procedure as at T0. These parameters were used to evaluate the disease alleviation with MAD as well as the therapeutic success in severe OSA-cases.

### Statistical analysis

Statistical analysis was performed using the software SPSS (version 29.0, IBM SPSS Statistics, Armonk, NY). Demographic and other clinical characteristics of the patients were reported for the total study collective as well as separately for the two groups to consider the influence of the baseline AHI/ h on disease alleviation. Univariate und multivariate linear regression analyses were performed for the total study collective as well as for the two groups to identify possible predictors of the AHI with MAD as well as of the absolute and relative AHI-reduction during MAD-treatment as means to determine disease alleviation. Multiple regression analysis was done only if the independent variables presented a *p* < 0.1. The determination coefficient *R²* was calculated to evaluate the effect size in case of a significant finding. Furthermore, the effect of categorical variables on disease alleviation was evaluated using absolute and relative frequencies and chi-squared test, whereas the influence of continuous parameters was analysed by applying independent, two-tailed t-tests. Due to the central limit theorem and the robustness of the t-test to moderate deviations from normality, parametric tests were applied. The level of significance was set at *p* < 0.05.

## Results

### Demographic characteristics

**Fig. 2:**
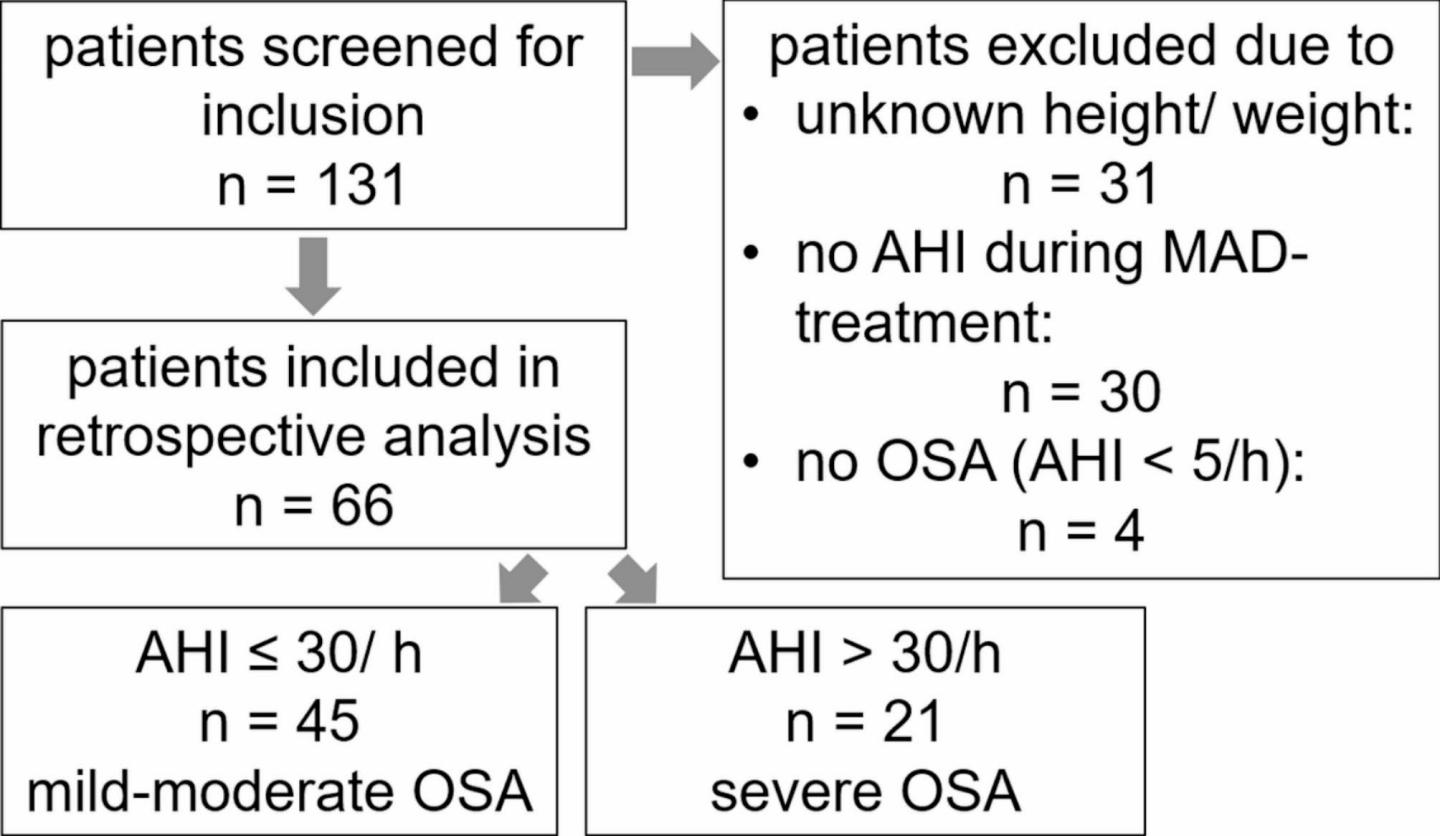
Flow-chart of the study population

After screening 131 patients, finally sixty-six patients with a mean age of 55 ± 11 years were included in this analysis (Fig. 2). The group with mild to moderate OSA consisted of 45 patients, whereas the severe OSA-group included 21 cases.

The demographic and baseline characteristics of the study population as well as of the two groups are presented in Table 1. More patients presented an AHI ≤ 30/ h, resulting in more mild to moderate compared to severe OSA-cases. Sex was not homogenously distributed among the total study population and the two groups, since male patients dominated. Body-mass index (BMI) was higher in severe OSA than in mild to moderate OSA, although this difference did not reach statistical significance (*p* = 0.126). The mean age of the participants was 55 years with a minimum increase in severe OSA, but without significant difference.

**Table 1:** Demographic and baseline characteristics of the total study population and the two groups at T0

variable	total (population)	AHI ≤ 30/ h	AHI > 30/ h	*p*-value
patients (*n*, %)	66, 100	45, 68	21, 32	-
male sex (%)	79	82	76	0.751
BMI ± SD (kg/m²)	27.9 ± 3.7	27.5 ± 3.3	28.9 ± 4.2	0.126
age ± SD (years)	55 ± 11	55 ± 10	56 ± 13	0.755

*n* = absolute numbers, % = relative frequency, SD = standard deviation, BMI = body mass index, T0 = before MAD-treatment.

The polygraphic and polysomnographic parameters assessed at T0 are shown in Table 2.By definition patients with AHI > 30/ h presented significantly higher AHI/ h, AI/ h and ODI/ h compared to patients with AHI ≤ 30/ h (*p* < 0.001). In line with this, the severity of OSA was significantly higher in patients with AHI > 30/ h (*p* < 0.001), as required by the allocation criteria. The amount of AHI in supine position/ h was similar between groups (*p* = 0.227), whereas the ratio between AHI in supine position and AHI was significantly higher in mild to moderate OSA-cases compared to severe OSA (*p* = 0.004).

**Table 2:** Poly(somno)graphic variables at baseline (T0) for the total study population and the two groups

variable		*n*	total population	AHI ≤ 30/h	AHI > 30/h	*p*-value
AHI/ h ± SD		66	25.0 ± 16.5	15.5 ± 6.5	45.3 ± 12.8	< 0.001*
AI/ h ± SD (*n* = 33 AHI ≤ 30/ h / *n* = 15 AHI > 30/ h)		48	13.9 ± 12.7	8.1 ± 6.0	26.5 ± 14.5	< 0.001*
AHI supine position/h (*n* = 28 AHI ≤ 30/ h / *n* = 12 AHI > 30/ h)		40	43.8 ± 26.1	40.5 ± 28.5	51.5 ± 18.3	0.227
ODI/ h ± SD (*n* = 41 AHI ≤ 30/ h / *n* = 19 AHI > 30/ h)		60	21.0 ± 17.7	13.0 ± 6.5	38.3 ± 21.6	< 0.001*
OSA	AHI > 30/ h (*n*, %)	21	21, 31.8	-	21, 100	< 0.001*
	AHI 15–30/ h (*n*, %)	20	20, 30.3	20, 44	-	< 0.001*
	AHI 5–14/ h (*n*, %)	25	25, 37.9	25, 56	-	< 0.001*
AI/ AHI (%) (*n* = 33 AHI ≤ 30/ h, *n* = 15 AHI > 30/ h)		48	53.9 ± 24.0	52.6 ± 26.6	56.8 ± 17.6	0.576
AHI supine position/ AHI (%) (*n* = 28 AHI ≤ 30/h / *n* = 12 AHI > 30/h)		40	2.2 ± 1.4	2.6 ± 1.4	1.3 ± 0.5	0.004*
ZA/ A (%) (*n* = 39 AHI ≤ 30/ h, *n* = 15 AHI > 30/ h)		54	24.8 ± 27.2	25.5 ± 29.5	19.3 ± 20.1	0.459
ZA/ AH (%) (*n* = 40 AHI ≤ 30/ h, *n* = 15 AHI > 30/ h)		55	10.6 ± 13.0	10.8 ± 13.9	10.0 ± 10.5	0.837

*n* = absolute numbers, % = relative frequency, SD = standard deviation. * Statistically significant at *p* < 0.05

Cephalometric analysis was conducted in 59 patients (87%) at T0 and in 37 cases (56%) at T1. The values, taken at T0, are given in Table 3. In the sagittal direction patients with AHI > 30/ h had a significantly more retrognathic mandible (SNB, *p* = 0.047) and chin position (SNPg, *p* = 0.035), whereas other sagittal parameters were not significantly different between groups. Considering the vertical direction, in severe OSA-cases the mandible presented a significantly higher degree of clockwise rotation (ML-NSL, *p* = 0.042) and a significantly more vertical growth pattern (Compound angle, *p* = 0.042). Parameters of posterior airway space were similar in both groups.

**Table 3:** Cephalometric analysis at T0 for the total study population and the two groups

dimension	cephalometric variable	total population (*n* = 59)	AHI ≤ 30/ h (*n* = 39)	AHI > 30/ h (*n* = 20)	*p*-value
sagittal	SNA [°] ± SD	82.1 ± 3.9	82.3 ± 4.2	81.7 ± 3.2	0.567
	SNB [°] ± SD	79.0 ± 4.1	79.8 ± 4.0	77.5 ± 4.0	0.047*
	ANB [°] ± SD	3.1 ± 3.6	2.5 ± 4.0	4.2 ± 2.5	0.102
	SNPg [°] ± SD	80.4 ± 4.2	81.3 ± 4.0	78.9 ± 4.1	0.035*
vertical	ML-NSL [°] ± SD	29.5 ± 7.6	28.1 ± 6.4	32.3 ± 9.0	0.042*
	NL-NSL [°] ± SD	7.5 ± 2.7	7.3 ± 2.6	8.0 ± 2.9	0.346
	ML-NL [°] ± SD	22.0 ± 7.3	20.8 ± 5.9	24.3 ± 9.1	0.083
	Compound angle [°] ± SD	389.5 ± 7.6	388.0 ± 6.5	392.3 ± 9.0	0.042*
	Gonion angle [°] ± SD	123.8 ± 8.3	123.2 ± 7.4	124.9 ± 9.9	0.475
	Jarabak ratio [%] ± SD	70.7 ± 6.1	71.8 ± 5.4	68.7 ± 6.9	0.064
PAS	PASP1 [mm]	25.1 ± ± 3.3	25.3 ± 3.3	24.6 ± 3.4	0.468
	PASP2 [mm]	20.0 ± 4.6	19.5 ± 4.6	21.3 ± 4.6	0.156
	PASP3 [mm]	11.4 ± 3.6	12.0 ± 3.7	10.2 ± 3.0	0.072
	PASP4 [mm]	11.5 ± 3.2	12.0 ± 3.4	10.3 ± 2.5	0.055
	PASP5 [mm]	15.2 ± 5.2	16.1 ± 4.9	13.3 ± 5.3	0.050
	PASP6 [mm]	17.6 ± 4.0	18.3 ± 4.0	16.4 ± 3.8	0.103

PAS = posterior airway space. Statistical significance tested by parametric independent two-tailed t-test. * Statistically significant at *p* < 0.05

At T1 poly(somno)graphy was repeated and the according results are illustrated in Table 4. AHI was reduced with MAD by 26.1 ± 16.0 / h in severe OSA and by 9.6 ± 6.4 / h in cases with AHI ≤ 30/ h. Relative AHI reduction was 55.5 ± 27.4% and 57.9 ± 33.3% in severe and mild to moderate OSA, respectively. During treatment with MAD, the AHI/ h, AI/ h and ODI/ h as well as the absolute AHI-reduction were significantly higher in severe OSA-cases (*p* < 0.001), although the relative AHI-reduction was not significantly different between groups (*p* = 0.397). Although the difference in OSA severity between the two groups was statistically significant at T1, both groups exhibited an overall reduction compared to baseline (T0).

**Table 4:** Poly(somno)graphic variables for the total study collective and two groups at T1

variable		*n*	total population	AHI ≤ 30/ h	AHI > 30/ h	*p*-value
AHI with MAD/ h ± SD		66	10.2 ± 9.6	6.0 ± 4.0	19.1 ± 11.7	< 0.001*
∆AHI/ h ± SD		66	-14.8 ± 12.9	-9.6 ± 6.4	-26.1 ± 16.0	< 0.001*
∆AHI (%)		66	-57.2 ± 31.3	-57.9 ± 33.3	-55.7 ± 27.4	0.397
AI/ h ± SD (*n* = 33 AHI ≤ 30/ h / *n* = 16 AHI > 30/ h)		49	4.6 ± 7.0	2.3 ± 2.6	9.4 ± 10.3	< 0.001*
AHI in supine position/ h (*n* = 10 AH ≤ 30/ h / *n* = 2 AHI > 30/ h)		12	14.1 ± 14.6	9.6 ± 10.8	36.2 ± 10.9	0.066
ODI/ h ± SD (*n* = 44 AHI ≤ 30/ h/ *n* = 19 AHI > 30/ h)		63	9.8 ± 8.8	5.8 ± 3.7	19.1 ± 10.1	< 0.001*
OSA	AHI > 30/ h (*n*, %)	4	4, 6	-	4, 19	< 0.001*
	AHI 15–30/ h (*n*, %)	8	8, 12	-	8, 38	< 0.001*
	AHI 5–14/ h (*n*, %)	30	30, 46	23, 51	7, 33	< 0.001*
	AHI < 5/ h (*n*, %)	24	24, 39	22, 46	2, 10	< 0.001*

* Statistically significant at *p* < 0.05

### Predictors of disease alleviation with MAD - regression analysis

The relevant results of the univariate and multivariate linear regression analysis are presented in the Supplementary Tables 1–3. Statistical analyses of sagittal skeletal parameters (SNA, SNB, ANB, SNPg, Wits-Appraisal) revealed no significant reliable predictors for AHI at T1 or for absolute and relative AHI reduction. Therefore, they are not mentioned in the analyses. Furthermore, among the six parameters assessing the posterior airway space, only PASP1 presented statistical significance in few cases, so that the other five parameters are also not listed. Generally, among the vertical skeletal, dental and soft tissue parameters, only those were mentioned, that were identified to be a significant predictor of at least one of the three dependent variables measuring disease alleviation.

At T1, no skeletal cephalometric parameter significantly predicted AHI with MAD, neither in the total study population nor in the two groups. However, considering the dental parameters, there was a significant correlation between AHI with MAD on the one hand, and, on the other hand, the inclination of the lower incisors in severe OSA-cases, and the inclination of the upper incisors and the interincisal angle in mild to moderate OSA-patients. Furthermore, analysis of the posterior airway space revealed, that PASP1 significantly predicted AHI at T1 in patients with AHI > 30/ h. Other predictors of AHI with MAD were BMI and oxygen saturation below 90% in the total study collective, baseline AHI in the total population and mild to moderate OSA-cases, and the amount of central apneas related to all apneas in patients with AHI ≤ 30/ h. In the multivariate model, the predictors could explain only 39.5% of the variation, whereas in the two groups more variation could be explained as determined by the higher R² (0.443 AHI ≤ 30/ h, 0.517 AHI > 30/ h).

Concerning the absolute AHI-difference/ h, the baseline-AHI proved to be a significant predictor, regardless of the severity of OSA (*p* < 0.001), and a high baseline-AHI was associated with a bigger AHI reduction. In contrast, AHI in supine position and oxygen saturation below 90% only acted as a predictor of all and mild to moderate OSA-patients. The ratio ZA/ AH, however, significantly predicted absolute AHI-reduction only for patients with a baseline AHI > 30/ h. Among the cephalometric variables investigated, neither dental, nor posterior airway space–related ones showed significant correlation. In the total study collective, ML-NL, ML-NSL, compound angle and Gonion angle served as significant predictors with a negative correlation. The Jarabak ratio was identified as a predicting variable for the total population and for severe OSA-patients. Finally, the sagittal position of the lower lip was correlated with the absolute AHI-reduction in both groups, although the regression coefficients showed a high range. In the multivariate model, the significant predictors could explain 65.5%, 62.3% and 98.3% of variation for all, mild to moderate and severe OSA-patients, respectively.

Regarding the last dependent variable, the relative AHI-reduction, the least significant predictors could be identified with the linear regression analysis. ZA/ AH presented a significant positive correlation for the total study collective as well as for the two groups in both the univariate and the multivariate linear regression. Furthermore, applying a multivariate model, the significant predictors baseline-AHI and ML-NL could be identified in the groups AHI ≤ 30/ h and AHI > 30/ h, respectively. However, all of the significant predictors showed only small regressions coefficients and effect sizes. Applying the multivariate model, the predicting variables were able to explain 23.2%, 25.3% and 75.0% of the variation for all, mild to moderate and severe OSA-patients, respectively.

### Necessity of lateral cephalograms

Based on the above presented findings, only few cephalometric parameters were identified to significantly predict disease alleviation with MAD (Supplementary Tables 1–3). In general, the significant variables were PASP1, inclination of the front teeth, interincisal angle, ML-NSL, ML-NL, compound angle, Gonion angle and Jarabak ratio.

### Reduction of apnea-hypopnea burden in severe OSA

To demonstrate the effect of different parameters on the evaluation of therapeutic success of MAD, two examples, representing a mild and a severe OSA-patient, were made, which are illustrated in Table 5.

**Table 5:** Apnea-hypopnea burden reduction in two individual patients with mild and severe obstructive sleep apnea

Dependent variable	AHI-reduction from 10/h to 5/h (AHI ≤ 30/ h)	AHI-reduction from 50/h to 25/h (AHI > 30/ h)
1. Baseline-AHI/ h	10.0	50.0
2. AHI with MAD/ h	5.0	25.0
3. AHI (%)	-50.0	-50.0
4. ΔAHI/ h	-5.0	-25.0

Measurements of two individual patients, representing mild (AHI ≤ 30/ h) and severe OSA (AHI > 30/ h), to demonstrate the effect of different parameters on the evaluation of treatment success of MAD with respect to the suppression of apneas and hypopneas

While the residual AHI on MAD is higher in the patient with severe OSA compared to the patient with mild OSA, the relative reduction of the apnea-hypopnea burden (AHI, %) is identical and the absolute apnea-burden reduction is 5-fold higher in the patient with severe OSA.

## Discussion

As shown by the descriptive analysis our study population was representative of OSA-patients with a higher prevalence of male and obese patients [3]. At baseline, severe cases showed a significantly more retrognathic and posteriorly rotated mandible, although such information does not precisely explain OSA-severity [23].

### Predictors of disease alleviation of OSA with MAD

Regression analyses identified several predictors of disease alleviation, but their influence varied depending on OSA severity and dependent variables.

Among the dependent variables, the highest R² was observed in the regression models for absolute AHI-reduction/ h (total study population 0.655, AHI ≤ 30/ h 0.623, AHI > 30/ h 0.983), indicating strong correlations [24]. In contrast, only a moderate and weak correlation were achieved for AHI/ h at T1 (R² = 0.395) and relative AHI reduction (R² = 0.232), respectively. Hence, the variables assessed did not predict relative AHI-decrease in a clinically relevant extent, especially not in the total study population and in the group AHI < 30/ h.

Baseline-AHI was statistically significant correlated with all dependent variables, although only in some groups. A high baseline-AHI was associated with a bigger absolute AHI reduction, as shown earlier [25]. Furthermore, baseline-AHI presented a positive correlation with AHI at T1. Burlon et al., however, found that baseline-AHI did not serve as a significant predictor of successful MAD-treatment [26]. This contradiction might be explained by another definition of success, i.e. an AHI-reduction by more than 50% and atherapeutic AHI/ h of < 10. Another poly(somno)graphic predictor was low oxygen saturation, being associated with higher AHI at T1. However, this correlation was insignificant in separated OSA-groups.

Among clinical and demographic parameters, BMI significantly predicted disease alleviation. The small (R² 0.076) positive correlation with AHI/ h at T1 indicated less success in obese patients, although only in the total population. Similarly, Buiret et al. found a significant negative correlation between BMI and relative AHI change [25]. As in the study of Chen et al. [27], sex and age did not present a significant correlation in our study, whereas others identified age as a predictor of successful treatment [16]. Different study populations and success definitions might explain this contrast.

The relevance of cephalometric parameters as predictors of disease alleviation appeared negligible. Systematic reviews report limited and partly contradictory evidence [28, 29]. Despite described correlations between OSA and some cephalometric parameters, heterogeneity of the available studies does not allow for generalised statements [22]. Our results showed significant correlations between some cephalometric parameters and disease alleviation: vertical growth pattern, hyperdivergent jaw bases and a posteriorly rotated mandible were associated with a bigger absolute AHI reduction. Other studies found a positive correlation between MAD-success and brachycephal growth pattern [30] or anterior rotation of the mandible [28]. Despite significant correlations between dental and PAS-related cephalometric variables with AHI at T1, R² were small and partly insignificant.

### Indication of lateral cephalograms for prediction of disease alleviation

Cephalometric variables analysed were significantly associated with outcome measurements of disease alleviation with MAD (PASP1, UK1/ML, interincisal angle, Labr-Inf-EL, ML-NSL, ML-NL, compound angle, Gonion angle, Jarabak ratio). Despite some statistical significance, R² was small, suggesting that pre-treatment lateral cephalograms are not indicated to estimate the treatment success in advance. Similarly, a systematic review concluded that the available evidence does not advocate cephalometric variables as predictors of MAD-success [29].

### Therapeutic success as disease alleviation with MAD in severe OSA-cases

Currently, MAD is considered as second line therapy only in specific cases (BMI < 30 kg /m² and AHI ≤ 30/ h) [13]. However, applying these criteria many OSA patients would be prevented from disease alleviation with MAD. Data highlighting the benefit of MAD especially in severe OSA are missing. Hence, this study was intended and able to show that even severe OSA patients experience noticeable disease alleviation with MADs. A high baseline-AHI was associated with higher absolute AHI reduction, indicating a successful disease alleviation of OSA even in severe OSA, despite the higher AHI at T1. Often, treatment success is the reduction of AHI by at least 50% o to AHI < 5/ h [27, 31]. Alternatively, AHI reduction by 75 − 25% [32], or a derease of AHI to < 20/ h [33] or to < 10/ h [34–36] is considered as good treatment response. Other outcome measures of interest for disease alleviation include the respiratory disturbance index (RDI) [15], the pre- to post-treatment comparison of subjectively reported symptoms or comorbidities [27] or changes in questionnaires like the ESS [27]. Here, the frequently used parameter absolute AHI reduction was analysed to enable comparisons with other studies. We observed a total mean AHI reduction/ h of 14.8 ± 12.9, which is similar to Buiret et al. (14.9 ± 11.9) [25]. According to our results and recent literature [37], absolute AHI decrease was higher in severe OSA. In contrast, in cases with low baseline-AHI the relative AHI difference achieved by MAD may overestimate the therapeutic success and vice versa. In our study, MAD reduced AHI by more than 50% in both groups, indicating a relevant disease alleviation. However, AHI/ h at T1 in severe OSA was higher than five, which would be regarded as treatment failure according to the success definition of AHI < 5/ h. This observation explains, why in severe OSA MAD is only suggested if CPAP is not possible [38]. However, when MAD is as an alternative for CPAP, the relative and especially the high absolute AHI reduction overweigh the residual AHI at T1. Moreover, in such cases any AHI reduction is an improvement. Therefore, providing a good selection of compliant patients, disease alleviation in severe OSA with MAD appears successful. Despite the effectiveness of MAD in OSA, potential side effects must be considered during MAD-treatment, because it may lead to inter- and intraarch differences [39] or functional complaints [27].

### Limitations

The retrospective design of the present investigation resulted in a sex imbalance with more males within the study cohort, which however, was in accordance with recent literature [3]. Another limitation was the upright standing position of lateral cephalograms, which neglects a potential backward movement of the mandible by gravity during sleep. Moreover, despite some correlations between lateral cephalograms and cone-beam computed tomography (CBCT) [40, 41], two-dimensional imaging does not precisely evaluate the three-dimensional upper airway volume, as sagittal measurements do not sufficiently correlate with cross-sectional ones [42]. The correlations between lateral cephalograms and CBCTs vary depending on the localisation in the upper airway [43]. Furthermore, airway measurements differ between inhalation and exhalation [44, 45]. However, three-dimensional radiographs cannot be justified as a routine diagnostic procedure due to the higher radiation exposure. Furthermore, the present study results might be hampered by solely investigating one MAD type, as MAD-effects may vary depending on the type of MAD [46, 47]. However, our study design ensured the absence of confounders due to MAD type. Our study is limited to a follow-up period of up to 6 months. This time was chosen to maximise the number of patients retrospectively included. However, future studies should extend the observation period to detect long-term effects, including side effects. It must be acknowledged that the sample size was small and hence results must be interpreted with caution. Finally, other factors, such as the neck circumference [16, 48, 49], the consumption of alcohol [49] and position dependency [31] were not addressed in the present study.

## Conclusions

The results of the present study show that disease alleviation with MAD in mild to moderate and severe OSA can be predicted by several factors. The strongest predictor identified was baseline-AHI, whereas lateral cephalograms did not provide a clinical relevant predictive value. Hence, within the limitations of this study, these radiographs are not indicated for predicting disease alleviation, but might still be justified, e.g. to evaluate side effects of MAD-treatment. Finally, a successful disease alleviation with MAD was shown also in severe OSA, suggesting MAD is a good alternative in cases of CPAP intolerance.

## Electronic supplementary material

Below is the link to the electronic supplementary material.

Supplementary Material 1

## Data Availability

No datasets were generated or analysed during the current study.

## Declarations

### Clinical trial number

Clinical trial number not applicable.’

## Abbreviations

MAD: Mandibular advancement device
OSA: Obstructive sleep apnea
AHI/ h: Apnea-hypopnea-index/ hour
AI/ h: Apnea index/ hour
ODI/ h: Oxygen saturation index/ hour
ZA: Central apnea
A: All apneas
AH: All apneas and hypopneas
CPAP: Continuous airway pressure
BMI: Body mass index
ESS: Epworth Sleepiness Scale
TAP: Thornton Adjustable Positioner
PSG: Polysomnography
PG: Polygraphy
CBCT: cone-beam computed tomography
