# Supplementary material for: Predictors of disease alleviation with mandibular advancement devices in obstructive sleep apnea: a retrospective cohort study
Source: Head Face Med. 2025 Apr 14;21:26. doi: 10.1186/s13005-025-00504-x (PMC11995498; doi:10.1186/s13005-025-00504-x)
Supplement: Supplementary file 1 — Supplementary Material 1 [file 13005_2025_504_MOESM1_ESM.docx]

| **Supplementary Material “ Predictors of disease alleviation with mandibular advancement devices in obstructive sleep apnea: a retrospective cohort study”** | | | | | | |
| --- | --- | --- | --- | --- | --- | --- |
|  |  |  | | |  | |
| **Table 1:** Predictors of AHI with MAD (T1) for the total study population and the subgroups. | | | | | | |
| **Dependent: AHI T1/ h** |  | univariate | | | Multivariate model:  total: *p* < 0.001; R² = 0.395  AHI ≤ 30/ h: *p* < 0.001; R² = 0.443  AHI > 30/ h: *p* = 0.008; R² = 0.517 | |
| independent |  | coefficient (95 % CI) | *p*-value | R² | coefficient (95 % CI) | *p*-value |
| BMI | total | 0.72 (0.09; 1.15) | **0.026*** | 0.076 | 0.18 (-0.37; 0.73) | 0.517 |
|  | AHI ≤ 30/ h | 0.09 (-0.29; 0.46) | 0.635 | 0.005 |  |  |
|  | AHI > 30/ h | 0.86 (-0.42; 2.13) | 0.176 | 0.094 |  |  |
| sex | total | -0.83 (-6.79; 5.14) | 0.783 | 0.001 | - | - |
|  | AHI ≤ 30/ h | -2.78 (-5.88; 0.33) | 0.078 | 0.070 |  |  |
|  | AHI > 30/ h | -0.46 (-13.37; 12.46) | 0.942 | 0.000 |  |  |
| age | total | -0.01 (-0.23; 0.21) | 0.942 | 0.000 | - | - |
|  | AHI ≤ 30/ h | 0.06 (-0.07; 0.18) | 0.357 | 0.020 |  |  |
|  | AHI > 30/ h | -0.14 (-0.56; 0.28) | 0.502 | 0.024 |  |  |
| AHI at T0 | total | 0.36 (0.25; 0.48) | **<0.001*** | 0.391 | 0.35 (0.23; 0.47) | **<0.001*** |
|  | AHI ≤ 30/ h | 0.21 (0.03; 0.39) | **0.027 *** | 0.109 | 0.14 (-0.04; 0.32) | 0.114 |
|  | AHI > 30/ h | 0.13 (-0.30; 0.57) | 0.526 | 0.021 |  |  |
| AHI supine | total | 0.04 (-0.05; 0.13) | 0.409 | 0.018 |  |  |
|  | AHI ≤ 30/ h | 0.00 (-0.06; 0.06) | 0.952 | 0.000 |  |  |
|  | AHI > 30/ h | 0.05 (-0.30; 0.40) | 0.746 | 0.011 |  |  |
| ZA/ AH | total | 0.12 (-0.10; 0.33) | 0.282 | 0.022 |  |  |
|  | AHI ≤ 30/ h | 0.12 (0.04; 0.21) | **0.007*** | 0.176 | 0.11 (0.023; 0.19) | **0.007*** |
|  | AHI > 30/ h | 0.18 (-0.44; 0.80) | 0.539 | 0.030 |  |  |
| SpO_2_ < 90 % | total | 0.50 (0.32; 0.67) | **< 0.001*** | 0.507 |  |  |
|  | AHI ≤ 30/ h | 0.32 (-0.14; 0.78) | 0.158 | 0.089 |  |  |
|  | AHI > 30/ h | 0.28 (-0.13; 0.68) | 0.152 | 0.239 |  |  |
| ML-NL | total | -0.13 (-0.50; 0.24) | 0.486 | 0.009 |  |  |
|  | AHI ≤ 30/ h | -0.12 (-0.34; 0.11) | 0.308 | 0.028 |  |  |
|  | AHI > 30/ h | -0.56 (-1.15; 0.04) | 0.065 | 0.177 | -0.41 (-0.90; 0.09) | 0.103 |
| NL-NSL | total | 0.71 (-0.25; 1.68) | 0.145 | 0.037 |  |  |
|  | AHI ≤ 30/ h | 0.24 (-0.27; 0.74) | 0.354 | 0.023 |  |  |
|  | AHI > 30/ h | 0.72 (-1.32; 2.76) | 0.468 | 0.030 |  |  |
| ML-NSL | total | -0.03 (-0.38; 0.33) | 0.883 | 0.000 |  |  |
|  | AHI ≤ 30/ h | -0.06 (-0.27; 0.15) | 0.573 | 0.009 |  |  |
|  | AHI > 30/ h | -0.49 (-1.11; 0.12) | 0.108 | 0.137 |  |  |
| Compound angle | total | -0.02 (-0.38; 0.33) | 0.896 | 0.000 |  |  |
|  | AHI ≤ 30/ h | -0.05 (-0.27; 0.16) | 0.614 | 0.007 |  |  |
|  | AHI > 30/ h | -0.49 (-1.11; 0.12) | 0.108 | 0.137 |  |  |
| Jarabak ratio | total | 0.04 (-0.40; 0.49) | 0.851 | 0.001 |  |  |
|  | AHI ≤ 30/ h | 0.11 (-0.15; 0.36) | 0.388 | 0.021 |  |  |
|  | AHI > 30/ h | 0.58 (-0.23; 1.39) | 0.149 | 0.112 |  |  |
| Gonion angle | total | -0.01 (-0.59; 0.57) | 0.971 | 0.000 |  |  |
|  | AHI ≤ 30/ h | 0.10 (-0.20; 0.41) | 0.490 | 0.013 |  |  |
|  | AHI > 30/ h | -0.74 (-1.89; 0.41) | 0.193 | 0.092 |  |  |
| UK1/ML | total | 0.20 (-0.13; 0.53) | 0.225 | 0.027 |  |  |
|  | AHI ≤ 30/ h | -0.07 (-0.22; 0.09) | 0.387 | 0.021 |  |  |
|  | AHI > 30/ h | 0.66 (0.00; 1.31) | **0.049*** | 0.198 | 0.63 (0.08; 1.12) | **0.029*** |
| OK1/NA [°] | total | -0.07 (-0.23; 0.09) | 0.371 | 0.014 |  |  |
|  | AHI ≤ 30/ h | -0.18 (-0.32; -0.03) | **0.016*** | 0.146 |  |  |
|  | AHI > 30/ h | -0.09 (-0.31; 0.13) | 0.403 | 0.039 |  |  |
| Interincisal angle | total | 0.07 (-0.12; 0.26) | 0.480 | 0.009 |  |  |
|  | AHI ≤ 30/ h | 0.11 (0.03; 0.20) | **0.010*** | 0.172 | 0.10 (0.02; 0.17) | **0.012*** |
|  | AHI > 30/ h | -0.01 (-0.41; 0.40) | 0.969 | 0.000 |  |  |
| Labr-Inf-EL | total | 0.10 (-0.65; 0.84) | 0.801 | 0.001 |  |  |
|  | AHI ≤ 30/ h | -0.03 (-0.39; 0.33) | 0.876 | 0.001 |  |  |
|  | AHI > 30/ h | -0.48 (-2.36; 1.40) | 0.598 | 0.016 |  |  |
| PASP1 | total | -0.74 (-1.51; 0.02) | 0.055 | 0.061 |  |  |
|  | AHI ≤ 30/ h | -0.03 (-0.43; 0.37) | 0.886 | 0.001 |  |  |
|  | AHI > 30/ h | -1.60 (-3.17; -0.03) | **0.046*** | 0.204 | -1.57 (-2.89; -0.25) | **0.023*** |

Uni- and multivariate linear regression analysis, * statistically significant at *p* < 0.05.

| **Table 2:** Predictors of absolute AHI reduction/ h for the total study population and the subgroups. | | | | | | |
| --- | --- | --- | --- | --- | --- | --- |
| **Dependent: ∆AHI/ h (absolute)** |  | univariate | | | Multivariate model:  total: *p* < 0.001; R² = 0.655  AHI ≤ 30/ h: *p* < 0.001 ; R² = 0.623  AHI > 30/ h: *p* = 0.035; R² = 0.983 | |
| independent variable |  | coefficient (95 % CI) | *p*-value | R² | coefficient (95 % CI) | *p*-value |
| BMI | total | -0.84 (-1,69; 0,02) | 0.056 | 0.056 | 0.09 (-0.48; 0.67) | 0.743 |
|  | AHI ≤ 30/ h | -0.06 (-0.66; 0.53) | 0.834 | 0.001 |  |  |
|  | AHI > 30/ h | -0.97 (-2.74; 0.80) | 0.264 | 0.065 |  |  |
| sex | total | -0.44 (-8.50; 7.62) | 0.914 | 0.000 | - | - |
|  | AHI ≤ 30/ h | 3.11 (-1.90; 8.12) | 0.217 | 0.035 |  |  |
|  | AHI > 30/ h | -2.82 (-20.42; 14.79) | 0.741 | 0.006 |  |  |
| age | total | 0.06 (-0.23; 0.35) | 0.687 | 0.003 | - | - |
|  | AHI ≤ 30/ h | -0.11 (-0.31; 0.09) | 0.272 | 0.028 |  |  |
|  | AHI > 30/ h | 0.33 (-0.23; 0.89) | 0.236 | 0.073 |  |  |
| AHI at T0 | total | -0.64 (-0.75; -0.53) | **<0.001*** | 0.666 | -0.58 (-0.71; 0.45) | **< 0.001*** |
|  | AHI ≤ 30/ h | -0.79 (-0.98; -0.61) | **< 0.001*** | 0.645 | -0.61 (-0.86; -0.36) | **< 0.001*** |
|  | AHI > 30/ h | -0.87 (-1.30; -0.43) | **< 0.001*** | 0.476 | -0.95 (-1.63; -0.26) | **0.027*** |
| AHI supine | total | -0.17 (-0.30; -0.04) | **0.014*** | 0.148 |  |  |
|  | AHI ≤ 30/ h | -0.12 (-0.20; -0.04) | **0.004*** | 0.283 |  |  |
|  | AHI > 30/ h | -0.10 (-0.58; 0.39) | 0.674 | 0.018 |  |  |
| ZA/AH | total | 0.22 (-0.03; 0.46) | 0.089 | 0.053 |  |  |
|  | AHI ≤ 30/ h | 0.101 (-0.09; 0.20) | 0.445 | 0.015 |  |  |
|  | AHI > 30/ h | 0.91 (0.09; 1.72) | **0.032*** | 0.308 |  |  |
| SpO_2_ < 90 % | total | -0.32 (-0.55; -0.09) | **0.008*** | 0.199 |  |  |
|  | AHI ≤ 30/ h | -0.48 (-0.94; -0.01) | **0.045*** | 0.171 |  |  |
|  | AHI > 30/ h | -0.16 (-0.80; 0.48) | 0.576 | 0.041 |  |  |
| ML-NL | total | -0.60 (-1.03; -0.17) | **0.007*** | 0.119 | -0.32 (-0.61; -0.03) | **0.033*** |
|  | AHI ≤ 30/ h | -0.02 (-0.35; 0.32) | 0.918 | 0.000 |  |  |
|  | AHI > 30/ h | -0.72 (-1.51; 0.08) | 0.075 | 0.165 |  |  |
| NL-NSL | total | -0.06 (-1.28; 1.16) | 0.924 | 0.000 |  |  |
|  | AHI ≤ 30/ h | 0.52 (-0.21; 1.26) | 0.156 | 0.054 |  |  |
|  | AHI > 30/ h | -0.16 (-2.91; 2.69) | 0.905 | 0.001 |  |  |
| ML-NSL | total | -0.56 (-0.97; -0.15) | **0.009*** | 0.114 |  |  |
|  | AHI ≤ 30/ h | 0.07 (-0.24; 0.39) | 0.635 | 0.006 |  |  |
|  | AHI > 30/ h | -0.75 ( -1.55; 0.05) | 0.065 | 0.177 |  |  |
| Compound angle | total | -0.56 (-0.98; -0.15) | **0.008*** | 0.117 |  |  |
|  | AHI ≤ 30/ h | 0.06 (-0.25; 0.37) | 0.687 | 0.005 |  |  |
|  | AHI > 30/ h | -0.75 (-1.55; 0.05) | 0.065 | 0.177 | -0.83 (-1.75; -0.10) | 0.061 |
| Jarabak ratio | total | 0.64 (0.10; 1.16) | **0.019*** | 0.095 |  |  |
|  | AHI ≤ 30/ h | -0.11 (-0.48; 0.26) | 0.551 | 0.010 |  |  |
|  | AHI > 30/ h | 0.95 (-0.10; 1.99) | **0.072*** | 0.168 |  |  |
| Gonion angle | total | -0.71 (-1.40; -0.02) | **0.044*** | 0.070 |  |  |
|  | AHI ≤ 30/ h | -0.09 (-0.53; 0.35) | 0.693 | 0.004 |  |  |
|  | AHI > 30/ h | -1.21 (-2.70; 0.28) | 0.105 | 0.139 |  |  |
| UK1/ML | total | 0.23 (-0.17; 0.64) | 0.253 | 0.024 |  |  |
|  | AHI ≤ 30/ h | 0.02 (-0.22; 0.26) | 0.879 | 0.001 |  |  |
|  | AHI > 30/ h | 0.79 (-0.11; 1.68) | 0.080 | 0.160 | 0.33 (-0.64; 1.30) | 0.284 |
| OK1/NA [°] | total | -0.14 (-0.33; 0.05) | 0.154 | 0.035 |  |  |
|  | AHI ≤ 30/ h | 0.04 (-0.18; 0.27) | 0.701 | 0.004 |  |  |
|  | AHI > 30/ h | -0.14 (-0.42; 0.15) | 0.335 | 0.052 |  |  |
| Interincisal angle | total | 0.04 (-0.20; 0.28) | 0.726 | 0.002 |  |  |
|  | AHI ≤ 30/ h | 0.02 (-0.22; 0.26) | 0.879 | 0.001 |  |  |
|  | AHI > 30/ h | 0.07 (-0.47; 0.60) | 0.792 | 0.004 |  |  |
| Labr-Inf-EL | total | -0.43 (-1.35; 0.49) | 0.348 | 0.016 |  |  |
|  | AHI ≤ 30/ h | 0.60 (0.12; 1.09) | **0.016*** | 0.152 | 0.15 (-0.19; 0.48) | 0.372 |
|  | AHI > 30/ h | -2.49 (-4.69; -0.30) | **0.028*** | 0.240 |  |  |
| PASP1 | total | -0.06 (-1.04; 0.92) | 0.900 | 0.000 |  |  |
|  | AHI ≤ 30/ h | -0.43 (-0.99; 0.13) | 0.127 | 0.059 |  |  |
|  | AHI > 30/ h | 0.05 (-2.29; 2.40) | 0.963 | 0.000 |  |  |

Uni- and multivariate linear regression analysis, * statistically significant at *p* < 0.05.

| **Table 3:** Predictors of relative AHI-reduction/ h for the total study population and the subgroups. | | | | | | |
| --- | --- | --- | --- | --- | --- | --- |
| **Dependent: relative AHI reduction (%)** |  | univariate | | | Multivariate model:  total: R² = 0.232  AHI ≤ 30/ h: *p* < 0.001; R² = 0.253  AHI > 30/ h: *p* = 0.002; R² = 0.750 | |
| independent variable |  | coefficient (95 % CI) | *p*-value | R² | coefficient (95 % CI) | *p*-value |
| BMI | total | 0.01 (-0.02; 0.03) | 0.639 | 0.003 |  |  |
|  | AHI ≤ 30/ h | 0.00 (-0.03; 0.03) | 0.927 | 0.000 |  |  |
|  | AHI > 30/ h | 0.01 (-0.02; 0.04) | 0.545 | 0.020 |  |  |
| sex | total | -0.01 (-0.29; 0.10) | 0.324 | 0.015 | - | - |
|  | AHI ≤ 30/ h | -0.12 (-0.38; 0.15) | 0.380 | 0.018 |  |  |
|  | AHI > 30/ h | -0.07 (-0.37; 0.23) | 0.630 | 0.012 |  |  |
| age | total | 0.00 (-0.01; 0.01) | 0.770 | 0.001 | - | - |
|  | AHI ≤ 30/ h | 0.00 (-0.01; 0.01) | 0.649 | 0.005 |  |  |
|  | AHI > 30/ h | 0.00 (-0.01; 0.01) | 0.924 | 0.000 |  |  |
| AHI at T0 | total | 0.00 (-0.01; 0.00) | 0.400 | 0.011 |  |  |
|  | AHI ≤ 30/ h | -0.01 (-0.03; 0.00) | 0.071 | 0.074 | -0.02 (-0.03; 0.00) | **0.034*** |
|  | AHI > 30/ h | -0.01 (-0.02; 0.00) | 0.235 | 0.073 |  |  |
| AHI supine | total | 0.00 (-0.01; 0.00) | 0.249 | 0.035 |  |  |
|  | AHI ≤ 30/ h | 0.00 (0.00; 0.00) | 0.274 | 0.046 |  |  |
|  | AHI > 30/ h | 0.00 (-0.01; 0.01) | 0.913 | 0.001 |  |  |
| ZA/ AH | total | 0.01 (0.00; 0.02) | **0.002*** | 0.171 | 0.01 (0.00; 0.01) | **0.004*** |
|  | AHI ≤ 30/ h | 0.01 (0.00; 0.02) | **0.012*** | 0.154 | 0.01 (0.00; 0.02) | **0.004*** |
|  | AHI > 30/ h | 0.01 (0.00; 0.03) | **0.024*** | 0.335 | 0.02 (0.01; 0.02) | **0.003*** |
| SpO_2_ < 90 % | total | 0.00 (0.00; 0.01) | 0.160 | 0.061 |  |  |
|  | AHI ≤ 30/ h | 0.00 (-0.03; 0.03) | 0.962 | 0.000 |  |  |
|  | AHI > 30/ h | 0.00 (-0.01; 0.01) | 0.724 | 0.016 |  |  |
| ML-NL | total | -0.01 (-0.02; 0.00) | 0.157 | 0.035 |  |  |
|  | AHI ≤ 30/ h | 0.00 (-0.02; 0.01) | 0.561 | 0.009 |  |  |
|  | AHI > 30/ h | -0.01 (-0.03; 0.00) | 0.070 | 0.171 | -0.01 (-0.03; 0.00) | **0.021*** |
| NL-NSL | total | 0.02 (0.00; 0.05) | 0.093 | 0.049 | 0.03 (0.00; 0.05) | 0.051 |
|  | AHI ≤ 30/ h | 0.02 (-0.01; 0.05) | 0.141 | 0.058 |  |  |
|  | AHI > 30/ h | 0.01 (-0.03; 0.00) | 0.070 | 0.171 |  |  |
| ML-NSL | total | 0.00 (-0.01; 0.01) | 0.445 | 0.010 |  |  |
|  | AHI ≤ 30/ h | 0.00 (-0.01; 0.01) | 0.958 | 0.000 |  |  |
|  | AHI > 30/ h | -0.01 (-0.03; 0.00) | 0.105 | 0.140 |  |  |
| Compound angle | total | 0.00 (-0.01; 0.01) | 0.452 | 0.010 |  |  |
|  | AHI ≤ 30/ h | 0.00 (-0.01; 0.01) | 0.952 | 0.000 |  |  |
|  | AHI > 30/ h | -0.01 (-0.03; 0.00) | 0.105 | 0.140 |  |  |
| Jarabak ratio | total | 0.01 (-0.01; 0.02) | 0.409 | 0.012 |  |  |
|  | AHI ≤ 30/ h | 0.00 (-0.02; 0.02) | 0.908 | 0.000 |  |  |
|  | AHI > 30/ h | 0.02 (0.00; 0.03) | 0.117 | 0.131 |  |  |
| Gonion angle | total | 0.00 (-0.02; 0.01) | 0.723 | 0.002 |  |  |
|  | AHI ≤ 30/ h | 0.00 (-0.02; 0.02) | 0.693 | 0.004 |  |  |
|  | AHI > 30/ h | -0.02 (-0.04; 0.01) | 0.189 | 0.094 |  |  |
| UK1/ ML | total | 0.00 (0.00; 0.01) | 0.343 | 0.016 |  |  |
|  | AHI ≤ 30/ h | 0.00 (-0.01; 0.01) | 0.845 | 0.001 |  |  |
|  | AHI > 30/ h | 0.01 (0.00; 0.03) | 0.079 | 0.162 | 0.01 (0.00; 0.03) | 0.061 |
| OK1/ NA [°] | total | 0.00 (-0.01; 0.00) | 0.136 | 0.039 |  |  |
|  | AHI ≤ 30/ h | -0.01 (-0.02; 0.00) | 0.186 | 0.047 |  |  |
|  | AHI > 30/ h | 0.00 (-0.01; 0.00) | 0.272 | 0.067 |  |  |
| Interincisal angle | total | 0.00 (0.00; 0.01) | 0.239 | 0.025 |  |  |
|  | AHI ≤ 30/ h | 0.00 (0.00; 0.01) | 0.188 | 0.048 |  |  |
|  | AHI > 30/ h | 0.00 (-0.01; 0.01) | 0.779 | 0.004 |  |  |
| Labr-Inf-EL | total | 0.01 (-0.01; 0.03) | 0.562 | 0.006 |  |  |
|  | AHI ≤ 30/ h | 0.02 (-0.01; 0.04) | 0.166 | 0.053 |  |  |
|  | AHI > 30/ h | -0.03 (-0.07; 0.01) | 0.174 | 0.100 |  |  |
| PASP1 | total | -0.01 (-0.03; 0.01) | 0.261 | 0.021 |  |  |
|  | AHI ≤ 30/ h | -0.01 (-0.03; 0.02) | 0.451 | 0.015 |  |  |
|  | AHI > 30/ h | -0.01 (-0.05; 0.03) | 0.529 | 0.022 |  |  |

Uni- and multivariate linear regression analysis, * statistically significant at *p* < 0.05.
